# Time-Domain Fluorescence Lifetime Imaging Techniques Suitable for Solid-State Imaging Sensor Arrays

**DOI:** 10.3390/s120505650

**Published:** 2012-05-02

**Authors:** David Day-Uei Li, Simon Ameer-Beg, Jochen Arlt, David Tyndall, Richard Walker, Daniel R. Matthews, Viput Visitkul, Justin Richardson, Robert K. Henderson

**Affiliations:** 1 Department of Engineering and Design, School of Engineering and Informatics, University of Sussex, Brighton BN1 9QT, UK; 2 Division of Cancer Research & Randall Division of Cell and Molecular Biophysics, Richard Dimbleby Department of Cancer Research, Guy's Campus, London SE1 1UL, UK; E-Mails: simon.ameer-beg@kcl.ac.uk (S.A.B.); viput.visitkul@kcl.ac.uk (V.V.); 3 SUPA, COSMIC, School of Physics and Astronomy, The University of Edinburgh, The King's Buildings, Mayfield Road, Edinburgh EH9 3JZ, Scotland, UK; E-Mail: j.arlt@ed.ac.uk; 4 Institute for Integrated Micro and Nano Systems, The School of Engineering, The University of Edinburgh, The King's Buildings, Mayfield Road, Edinburgh EH9 3JL, Scotland, UK; E-Mails: d.tyndall@ed.ac.uk (D.T.); richard.walker@ed.ac.uk (R.W.); justin.richardson@ed.ac.uk (J.R.); robert.henderson@ed.ac.uk (R.K.H.); 5 Queensland Brain Institute, University of Queensland, St. Lucia, QLD 4072, Australia; E-Mail: d.matthews1@eq.edu.au

**Keywords:** fluorescence lifetime imaging microscopy, FLIM, CMOS, single-photon avalanche diode, single-decay, multi-decay, time-domain FLIM, lifetime based sensing

## Abstract

We have successfully demonstrated video-rate CMOS single-photon avalanche diode (SPAD)-based cameras for fluorescence lifetime imaging microscopy (FLIM) by applying innovative FLIM algorithms. We also review and compare several time-domain techniques and solid-state FLIM systems, and adapt the proposed algorithms for massive CMOS SPAD-based arrays and hardware implementations. The theoretical error equations are derived and their performances are demonstrated on the data obtained from 0.13 μm CMOS SPAD arrays and the multiple-decay data obtained from scanning PMT systems. *In vivo* two photon fluorescence lifetime imaging data of FITC-albumin labeled vasculature of a P22 rat carcinosarcoma (BD9 rat window chamber) are used to test how different algorithms perform on bi-decay data. The proposed techniques are capable of producing lifetime images with enough contrast.

## 1. Introduction

Fluorescence based imaging techniques have become essential tools for a large variety of disciplines, ranging from cell-biology, medical diagnosis, and pharmacological development to physical sciences. Fluorescence lifetime imaging microscopy (FLIM) yields images with each pixel sensing the exponential decay rate instead of the intensity of the fluorescence from a fluorophore. It has the advantages of insensitivity to variations in excitation illumination, photon scattering and probe concentration, and it allows the resolution of different fluorophores (with different lifetimes) fluorescing at the same wavelength. FLIM can be applied to acquire more quantitative information about physiological parameters such as pH, Ca^2+^, pO_2_, *etc.* or image intracellular functions with Förster resonance energy transfer (FRET) techniques [1–3].

Both time-domain and frequency-domain instrumentation systems are available to acquire FLIM data. Here, we will focus on the recently proposed time-domain approaches and discuss how they can be applied to the latest solid-state sensor arrays for FLIM applications. For detailed discussions in frequency-domain FLIM systems, please see [4–7]. In a typical time-resolved FLIM experiment, the samples with fluorescent markers are illuminated by a pulsed laser and the time-correlated photons emitted from the markers are collected by detectors. Commercially available FLIM systems usually use photomultiplier tubes (PMT) or multiple channel plate (MCP) PMTs plus time-correlated single-photon counting cards (TCSPC) [8] or gated intensified/electron-multiplying CCDs to measure the lifetimes. The latest multi-channel PMT systems can significantly increase the imaging speed, but they still require image-scanning. For example, to avoid local heating and photobleaching, the pixel dwell time is set to be 15.25 μs and the sample is scanned hundreds of times, say 200 times, to accumulate enough photon counts. It will take 256 × 256 × 200 × 15.25 μs/*N*_p_ ∼ 200 s/*N*_p_ (*N*_p_ is the number of channels) to generate a 256 × 256 lifetime image. CCD based systems usually require coolers and/or high voltage supplies. Compared with CMOS compatible solutions, the above mentioned systems are expensive, bulky, and not user-friendly. Recent developments on CMOS-based FLIM systems can be found in [9–20]. Kawahito applies pin photodiodes whereas Shepard uses active pixel sensors, but both employed the 2-gate rapid lifetime determination (RLD) method [21] to calculate the lifetimes. Li *et al.* proposed several non-gating time-domain lifetime algorithms and demonstrated video-rate lifetime imaging [14,15] on single-photon avalanche diode (SPAD) plus in-pixel TCSPC arrays [22]. Unlike conventional CCD based sensors, a SPAD is a p-n junction reverse biased above the breakdown voltage to sustain the avalanche multiplication process triggered by photogenerated carriers. The transfer gain is so large that the output current from the SPAD can be easily converted into a digital signal without using complex front-end amplifiers deteriorating the signal-to-noise ratio (SNR). With such single photon sensitivity, SPADs are suitable for photon-starved applications such as single molecule detection [23,24], fluorescence lifetime measurements [13,20], optical range finding, optical fiber fault detection, and portable explosives sensing [25]. Recent developments of CMOS SPADs have shown significant improvements in the dead time [26], dark count [27], technology migration to advanced process [28] and pixel miniaturization [29], and quantum efficiency in the longer wavelength region [30]. It is expected high resolution CMOS SPAD arrays for ranging applications [31] will soon be applied to FLIM applications.

**Figure 1.**
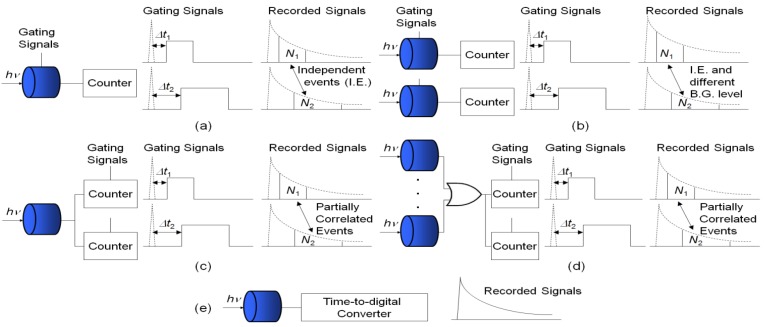
(**a**) Sequential acquisition in a pixel; (**b**) parallel acquisition in a super pixel (more than one detector in a pixel) or multi-channel gated optical intensifiers; (**c**) parallel acquisition with gated counters; (**d**) parallel acquisition with binned pixels and gate counters; (**e**) single-channel PMT+TCSPC or in-pixel SPAD+TCSPC acquisition.

Another bottleneck for high-speed lifetime imaging is lifetime calculation. Common FLIM systems usually use iterative linear or non-linear least square methods (LSM), such as Marquardt-Levenberg algorithms, to extract the lifetimes. Although this approach is accurate and suitable for analyzing multi-exponential decays, it is computationally time consuming which makes it unsuitable for real-time applications. It is desirable, therefore, to develop non-iterative simple algorithms to speed up the lifetime calculations while maintaining enough imaging quality. Compared with the LSM, iterative-free gating methods only require two time bins for single-exponential decays [9,13,21], four bins for bi-exponential decays [32,33] or eight bins for multi-exponential decays [34]. The hardware complexity is greatly reduced and the speed is much higher. There are different acquisition schemes for the gating methods. Figure 1(a) shows the traditional sequential acquisition in a pixel, where at least two sub-images are recorded sequentially at different delayed windows with respect to the excited laser pulses to extract the lifetime. The block ‘counter’ can contain front-end circuits, analog-to-digital converters and accumulators in conventional imaging systems or simply inverters and digital buffers in the latest CMOS SPAD systems. Chang and Mycek applied four time-gates to analyze single-exponential decay data [35]. This approach is slow and sensitive to motion artifacts unless the samples are stationary, and the recorded sub-images are uncorrelated. If a full fluorescence emission histogram is needed for detailed examinations, it will take a significant amount of time to record a large number of sub-images with different delay times [36].

Unlike the sequential acquisition scheme, some researchers applied ‘single-shot’ acquisition methods by applying multi-channel segmented gated optical intensifiers (GOI) [37] or focusing optically delayed images on different sections of the detector [38,39] to collect sub-images simultaneously. Video-rate FLIM has been demonstrated in GOI FLIM systems. This trades off spatial resolution for imaging speed. Similar to the sequential acquisition scheme, the detectors enabled by the gating signals only work ‘part-time’. The background level for the two detectors could be different, and the two recorded events are independent (uncorrelated).

Figure 1(c) shows a different acquisition scheme from above. The detector works fully, but with several gated counters collecting photons in different timing windows. The gated counter accumulates only when the leading edge of the detector output is inside its enable period. The gating signals for controlling the counters can be non-overlapping [36–40] or overlapping [41]. Gerritsen *et al.* applied several non-overlapping gated counters to confocal scanning systems [40]. The system can deal with multiple-decay data. The overlapping gating acquisition, denoted as ORLD, was first proposed by Chan *et al.* [41] and it was proven to be efficient by running Monte Carlo simulations, but the authors only suggested splitting the PMT signal and sending to two gated counters without actually carrying out the experiments probably because of the complexity and the cost of reconfiguring the existing acquisition systems. Later, Elangovan *et al.* proposed an overlapping multi-gated algorithm to analyze double-exponential decays and applied Monte Carlo simulations to find suitable experiment settings [32]. For the latest developed CMOS SPAD arrays, however, the ORLD is a good option considering the system complexity and photon efficiency (the photon events collected in the gated counters are correlated if the overlapped proportion is significant). We will for the first time derive the theoretical error equations and test the ORLD methods on the data obtained from a 0.13 μm CMOS SPAD array.

Stoppa's research team combined the acquisition schemes (b) and (c) by binning 4 and 25 CMOS SPADs, respectively, in a ‘super’ pixel and using gated counters to collect the photon events [11,12] as in Figure 1(d). Similar to the second gating scheme, this technique trades the spatial resolution for imaging speed. It provides faster acquisition in some sensing applications such as high throughput screening. They used non-overlapping time gates to collect the photons and used nonlinear LSM to extract the lifetimes.

Figure 1(e) shows a pixel with time-correlated single-photon counting (TCSPC) circuitry. The detector works fully, with a time-to-digital converter (TDC) to record the time tag of each photon. Compared with the hardware for time-gating acquisition, the complexity of the TCSPC systems is much higher. The latest commercial multi-channel systems only contain several PMTs and still require scanning. Recently we have demonstrated a 1024-channel, a 20 k-channel and a 100 Mphoton/s multi-channel FLIM systems with 0.13 μm CMOS SPAD plus in-pixel TDC (10-bit TCSPC) arrays [14–16,20]. The parallelism of the proposed systems can be fully exploited by implementing lifetime calculations in hardware [42,43]. Similar to the above-mentioned ‘single-shot’ systems, the imaging speed of the proposed cameras can operate over 100 f/s.

## 2. Time-Domain FLIM Data Analysis

For simplicity, we consider a single-exponential decay with a negligible impulse response function (IRF). Such assumptions allow a proper comparison of various fitting algorithms. Moreover, a single-exponential decay model is still enough to contrast different types of fluorophores. We will discuss the accuracy of FLIM algorithms and compare the performance of various algorithms on bi-exponential decays. Assume that the fluorescence decay function *f*(*t*) = *A*exp(−*t*/*τ*)u(*t*) with *τ* being the lifetime and u(*t*) the step function.

### 2.1. Gating Techniques

**Figure 2.**
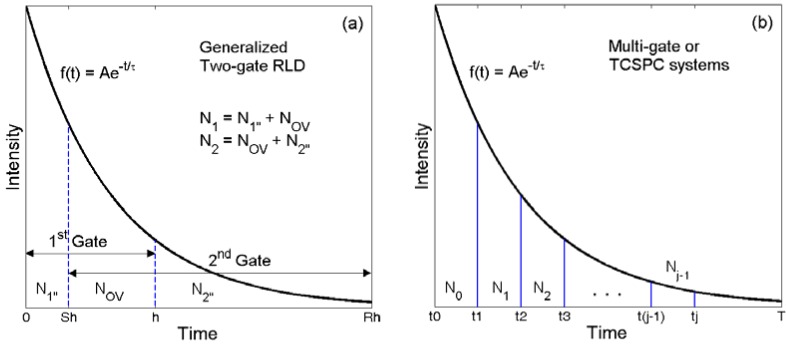
(**a**) Generalized two-gate rapid lifetime determination and (**b**) multi-gate or TCSPC acquisition.

Gating algorithms have been widely used in real-time FLIM systems owing to their simplicity and compactness in hardware implementations. The simplest 2-gate RLD (RLD-2) only uses two time gates of the same width. We adopt the *F* value introduced in [44] to quantify the performance of a FLIM algorithm. The *F* value is defined as *F* = *N_c_*^1/2^*σ_τ_*/*τ*, where *σ_τ_* is the standard deviation of repeated measurements of the lifetime value *τ* and *N_c_* the total count in the measurement window from *t* = 0 to *t* = *T*, the average total count $EN_{c}=\int_{0}^{T}f\left(t\right)dt=A\tau \left(1-e^{-T/\tau }\right)$. The *F* value for the RLD-2 can be derived from [21] and [44] as:

(1) $$F_{\text{RLD}}=\sqrt{N_{c}}{\frac{\sigma_{\tau }}{\tau }|}_{\text{RLD}}\sim \frac{x+1}{h\sqrt{x}},$$

where *x* = exp(−*h*/*τ*) with *h* being the gate width. The two gates can be generalized to be overlapping with unequal widths as shown in Figure 2, denoted as GRLD hereafter, where *N*_1_ (= *N*_1″_ + *N*_ov_) and *N*_2_ (= *N*_ov_ + *N*_2″_) are the total counts of the first (0 < *t* < *h*) and second (*Sh* < *t* < *Rh*) gates respectively. Chan *et al.* [41] used Monte Carlo simulations to get an optimized setting (*S* = 0.25, *R* = 12.25) and mentioned that the PMT signal could be split and sent to two gated integrators, but the experiments were not actually carried out. Their “optimized setting” were not based on the theoretical derivations, and we will present theoretically optimized settings that are different from their suggestions. We will also compare its performance with other time-domain algorithms. From the discussions in Section 1, the GRLD can be applied to different gating schemes shown in Figure 1(a–d). We divide these acquisition schemes into two categories. The first category is uncorrelated, where the overlapping parts are uncorrelated, denoted as UGRLD hereafter, because they are recorded in different snapshots or in different gated detectors. The photon events *N*_1_ and *N*_2_ are mutually independent and therefore uncorrelated. The second category is correlated, denoted as CGRLD hereafter, because the overlapping counts, *N*_ov_, collected by different gated integrators are from the same photon events. The *F*-values for the UGRLD and CGRLD can be derived directly from Equation (A5) in the Appendix (using similar derivation methods and notations with [45]) as:

(2) $$\begin{array}{l} F_{\text{UGRLD}}=\frac{\sigma_{\tau }}{\tau }\sqrt{N_{c}}=\frac{\tau h^{-1}\sqrt{k_{1}(x)}}{\left[\left(1-S\right)x^{S+1}+\left(R-1\right)x^{R+1}+Sx^{S}-Rx^{R}\right]},\\ k_{1}(x)=\left(1-x\right)\left(1-x^{R}\right)\left(x^{S}-x^{R}\right)\left(1-x+x^{S}-x^{R}\right),\\ F_{\text{CGRLD}}=\frac{\sigma_{\tau }}{\tau }\sqrt{N_{c}}=\frac{\tau h^{-1}\sqrt{k_{2}(x)}}{\left[\left(1-S\right)x^{S+1}+\left(R-1\right)x^{R+1}+Sx^{S}-Rx^{R}\right]},\\ k_{2}(x)=k_{1}(x)-2\left(1-x\right)\left(1-x^{R}\right)\left(x^{S}-x\right)\left(x^{S}-x^{R}\right). \end{array}$$

Equation (2) allows us to determine an optimized setting without resorting to Monte Carlo simulations. Moreover, setting *g*(*x*) = 0 in Equation (A2), we can obtain:

(3) $$\frac{x^{S}\left(1-x^{R-S}\right)}{1-x}=\frac{N_{2}}{N_{1}}\Rightarrow x^{S}\cdot \sum_{i=0}^{R-S-1}x^{i}=\frac{N_{2}}{N_{1}}\Rightarrow x{\left(\sum_{i=0}^{R-S-1}x^{i}\right)}^{1/S}={\left(N_{2}/N_{1}\right)}^{1/S},$$

where both *S*^−1^ and (*R*−*S*) are integers. It is easy to build a look-up table for Equation (3) on FPGAs and make the calculations much faster as in [15].

**Figure 3.**
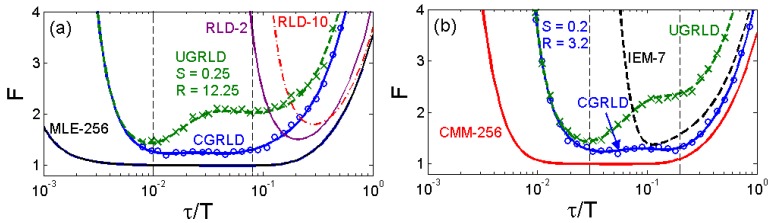

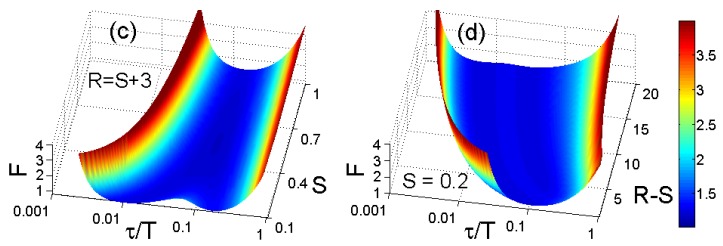
*F*-value *versus τ*/*T* plots of (**a**) previously reported 256 gate MLE, 2-gate RLD, 10-gate RLD (RLD-10), CGRLD/UGRLD with S = 0.25, R = 12.25, and (**b**) the proposed 256-gate CMM, 7-gate IEM, CGRLD/UGRLD with S = 0.2, R = 3.2. 3-D *F*-value plots of (**c**) *R*−*S* = 3 and (**d**) *S* = 0.2.

Some researchers used mult-gate RLD (RLD-*M*) to calculate the lifetimes [35,36,42]:

(4) $$\tau_{\text{RLD}-M}=\left[{\left(\sum_{j=0}^{M-1}t_{j}\right)}^{2}-M\sum_{j=0}^{M-1}t_{j}^{2}\right]/\left\{M\sum_{j=0}^{M-1}\left[t_{j}\ln\left(N_{j}\right)\right]-\sum_{j=0}^{M-1}t_{j}\sum_{j=0}^{M-1}\ln\left(N_{j}\right)\right\},$$

where *N_j_* is the count number in the *j*th time bin (*t_j_* < *t* < *t_j_*_+1_), *j* = 0, …, *M*-1. Similar to Equation (A2), an average lifetime is obtained from Equation (4) if the fluorescent emission is a multi-exponential decay. The theoretical error equation, Equation (4), only exists when the bins are equally spaced, and it was for the first time derived in [42] considering also the impact of timing jitters and later derived in [35] for a special case (*M* = 4) incorporating filtering techniques. Neglecting the impact of the TDC jitters, the *F*-value can be re-written from Equation (25) in [42] as:

(5) $$\begin{array}{c} F_{\text{RLD}-M}=\frac{6\tau }{M\left(M^{2}-1\right)h}\sqrt{\frac{1-x^{M}}{1-x}G\left(x\right)},x=\exp\left(-h/\tau \right),\\ G\left(x\right)={\left(x^{-1}-1\right)}^{-3}\left[{\left(M-1\right)}^{2}\left(x^{-M-2}-1\right)+\left(6-2M^{2}\right)\left(x^{-M-1}-x^{-1}\right)+{\left(M+1\right)}^{2}\left(x^{-M}-x^{-2}\right)\right]., \end{array}$$

where *h* is the bin width. Figure 3(a,b) shows the *F*-value curves in terms of the ratio of the lifetime over the measurement window for the previously reported and our center-of-mass method (CMM) [15], integration for lifetime extraction method (IEM) [42], Equations (1), (2) and (5). The 256-gate maximum likelihood estimation (MLE) offers the best precision with a very wide optimized window. RLD-2 has its best performance at *T* ∼ 4.8*τ* (or *h* = 2.4*τ*) [21], and it usually requires to know the lifetime range of the samples in advance for tuning a proper gate width. Theoretical (solid lines) and Monte Carlo simulation (marked with circles or crosses) results of the UGRLD and CGRLD show good agreement with Equation (2). For extracting single-decay lifetimes, the performance of RLD-*M* is *only* better than the other gating techniques in the region *T* ∼*τ* and increasing *M* over eight does not improve the precision any further. This conclusion contradicts the comment stating that the RLD-*M* is more precise than RLD-2 made in [35]. In their experiments, they applied four time gates on single-exponential decays. The advantage of using the multiple time gate technique, however, is its capability to image multi-exponential decays [11,34] using linear or nonlinear LSMs or to image bi-exponential decays using the fast algorithm proposed by Sharman and Periasamy [32,33]. The performance of UGRLD is worse than that of CGRLD. For the same acquisition time, the gated integrator acquisition schemes shown in Figure 1(c,d) provide a better precision than those in Figure 1(a,b). Chan *et al.* suggested an optimized setting (*S* = 0.25, *R* = 12.25) [41], where the best resolving window is roughly from *T* ∼ 12 to 100*τ* with the duty cycle of their lifetime measurements being comparatively low. Figure 3(c,d) show two *F*-value plots with (*R-S*) fixed and with *S* fixed respectively. For *R* = *S* + 3, a smaller *S* results in a wider optimized window allowing to resolve smaller lifetimes, but the minimum *F* value occurs at about *S* = 0.5. It is obvious that a bigger *R* with a proper *S* (around 0.2 to 0.7 considering the lifetime resolvability range) allows resolving lifetimes much less than the measurement window. The lower bound of the resolvable lifetime range, however, is limited by the full width at half maximum of the IRF, so choosing a much bigger *R* is not realistic. Instead of using the setting suggested by Chan *et al.*, we suggest an optimized setting (*S* = 0.2 and *R* = 3.2) from Equation (2) to provide a comparable performance with RLD-2 in *T* < 5*τ* while maintaining an enough resolvability up to *T* ∼ 50*τ*. For a laser repetition rate of 80 MHz, the minimum resolvable lifetime is about 200 ∼ 300 ps, in the same order of the timing jitters of the latest developed 0.13 μm CMOS SPADs [22]. The minimum *F*-value of the CGRLD is about 1.2 and its simplicity in hardware implementation is *promising* for massive sensor arrays. In Figure 3(b), the IEM-7 (7-gate IEM) [42] and CMM-256 (256-gate CMM) [15] are non-iterative algorithms suitable for TCSPC systems.

### 2.2. Non-Iterative Algorithms Suitable for TCSPC Systems

**Figure 4.**
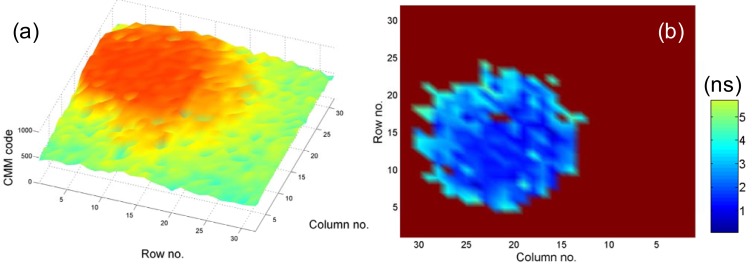
(**a**) (Video) Movie of CMM lifetime codes of fluorescent beads in a micro-channel at a video frame rate of 50 fps and (**b**) a single frame of the estimated lifetime video.

Although the gating methods introduced above can provide fast lifetime preview and are easy to implement, scientists still prefer to keep the raw timing data for detailed examinations on what really happens in the samples. For gating methods, recording raw timing data is not easy. To obtain a full histogram, a large number of snap shots at different time delays are acquired sequentially. It is very inefficient. To increase the raw data collecting speed, the number of gated integrators can be increased as in [34], but at a cost of system complexity. To enhance the raw data collection rate, we incorporated on-chip high-resolution TDCs into the CMOS SPAD arrays and built 1024-channel and 20k-channel “miniaturized PMT+TCSPC” prototypes [16]. The prototypes can operate in the RAW MODE for collecting the raw data and in the COARSE MODE for high-speed lifetime imaging preview. Due to the slow speed of the widely used iterative based least square methods, several time-domain FLIM algorithms suitable for TCSPC systems (Figure 1(e)) have been proposed to improve the imaging speed [42,43]. We proposed an innovative background-insensitive algorithm suitable for our SPAD prototypes, called IEM. The calculated lifetimes are:

(6) $$\tau_{\text{IEM}}=\frac{h\cdot \sum_{j=0}^{M-1}\left({\bar{C}}_{j}N_{j}\right)}{N_{0}-N_{M-1}},$$

where **C̄** = [1/3, 4/3, 2/3, …, 4/3, 1/3] from the Simpson's integration rule [42], *h* is the TDC bin width, and *N_j_* is the count number in the *j*th time bin. The precision and accuracy are detailed in [42]. Compared with commonly used RLD-2 and MLE, IEM is much less sensitive to the background noise [46] Moreover, the lifetime calculations are very simple and suitable for on-FPGA or on-chip implementations. On-FPGA real-time wide-field lifetime imaging has been successfully demonstrated on microfluidic mixing [14]. Figure 3(b) also shows an *F*-value curve for the 7-gate IEM with a total count of 1024 (IEM is a biased estimator and the quantization error which comes to effect at *τ* < 0.1*T* (for *M* = 7) is included in the *F*-value curve as in [42]. Without considering the impact of the background noise, the error performance of IEM-7 is similar to RLD-2. The effect of increasing *M* in IEM is similar to increasing *R* in GRLD; both cause their *F*-value curves to shift towards the smaller *τ*/*T* region. To further enhance the lifetime imaging speed on our SPAD prototypes, we proposed another hardware implementation algorithm based on center-of-mass methods. The calculated lifetime defined in [43] is re-written as:

(7) $$\tau_{\text{CMM}}\sim \frac{\int_{0}^{T}tf\left(t\right)dt}{\int_{0}^{T}f\left(t\right)dt}=\tau -\frac{Te^{-T/\tau }}{1-e^{-T/\tau }}$$

(8) $$\tau_{\text{CMM}}=\left(\frac{\sum_{i=1}^{N_{c}}{\bar{D}}_{i}}{N_{c}}+\frac{1}{2}\right)h,\tau =\Omega \left(\frac{\tau_{\text{CMM}}}{T}\right)\cdot T=\Omega \left[\frac{1}{M}\left(\frac{\sum_{i=1}^{N_{c}}{\bar{D}}_{i}}{N_{c}}+\frac{1}{2}\right)\right]\cdot Mh,$$

where *D̄_i_* is the 10-bit TDC output of the *i*th captured photon and Ω(·) is a 1D look-up table for calibrating the error term in Equation (7). Its precision and accuracy Equations were derived in [15], and its *F*-value curve (shown in Figure 3(b)) considering the accuracy term for 256-gate CMM with *N_c_* = 1,024 showing great photon effectiveness with a wide optimization range. We applied Equation (7) to achieve high-speed lifetime sensing [20,43] and imaging [15]. The lifetime calculations are mostly carried out on FPGAs. To demonstrate its performance, a widefield microscope was adapted to accommodate the SPAD array. A dilute solution of 10 μm fluorescent beads (G1000, Duke Scientific, Palo Alto, CA, USA) was used to simulate cells flowing through the system. The fluorescent emission was captured by the SPAD camera and the delay of every captured photon with respect to the laser pulses was calculated by the in-pixel TDC. The 10-bit CMM lifetime codes were generated from the FPGA through Equation (8), and the lifetimes were easily calculated with the characterization data of the TDC array. Figure 4(a) shows CMM codes at a video frame rate of 50 fps, and Figure 4(b) shows a single frame of the estimated lifetime video. The size of the bead is about 10 μm, and the speed of beads is 200 μm/s. The experimental settings are optimized for the TDC sub-array with the resolution *h* = 78 ps, Rows 1−16 [22], and the lifetime codes obtained from the other part (*h* = 160 ps, Rows 17−32) are normalized. During one frame time the bead moves approximately 4 μm, 40% of its width, producing non-trivial motion blur. The average lifetime is around 2 ns, in a good agreement with the lifetime obtained by using the burst integrated fluorescence lifetime (BIFL) measurement with a Becker & Hickl card (SPC850). The microscope was based on a Nikon Eclipse Ti-E inverted microscope platform. Illumination was via a polarization maintaining fibre to the Nikon TIRF attachment using a 4 W supercontinuum laser (Fianium SC400) to provide picosecond laser pulses at 20 MHz. Excitation wavelengths are selected using bandpass filters prior to launching into the fibre. In this instance a 488 nm bandpass filter was used (470 ± 22 nm, Chroma Inc, Bellows Falls, VT, USA) unless stated otherwise. Fluorescence lifetime images were acquired with a Nikon 40× Plan Fluor Objective (0.75 NA) using the 0.13 μm CMOS 32 × 32 SPAD+TDC chip [22] arranged in the image plane of the microscope side port. The microfluidic system consisted primarily of a Mitos T-Junction Chip (100 μm channel) connected via 1/16″ Teflon tubing to a Mitos P-pump (The Dolomite Centre, Royston, UK). The instrument response function (IRF) was measured and found to have a FWHM of 0.6 ns.

The analog version of Equation (7), denoted as analog mean-delay (AMD) hereafter, has been reported earlier using stand-alone components for lifetime sensing applications [47]. Recently, an AMD instrumentation considering the impact of IRFs and limited measurement window problems has been carried out almost at the same time with our digital CMM by Kim [48,49]. Their algorithms can work efficiently with low-cost single-channel PMTs, and it has been demonstrated in a confocal scanning microscope [50]. Padilla-Parra *et al.* also used Equation (7) to calculate the minimal fraction of interacting donor (MFD) in florescence resonance energy transfer (FRET) experiments [51,52]. The measurement window of the MFD, however, should be much larger than the largest lifetime component; otherwise the contrast capability would be lost, similar to the problems revealed in [15] and [49]. By automatically calibrating such problems, the MFD can be easily applied to our SPAD systems for FRET applications as well. Recently, a Bayesian FLIM analysis method was proposed, and it outperformed the commonly used MLE and LSM especially on noisy single-exponential data sets [53,54]. This algorithm is promising especially in single-cell cytometry. The Bayesian methods for multi-exponential decays are under development.

### 2.3. Error Performance of FLIM Algorithms on Data Collected by CMOS SPAD Arrays

**Figure 5.**
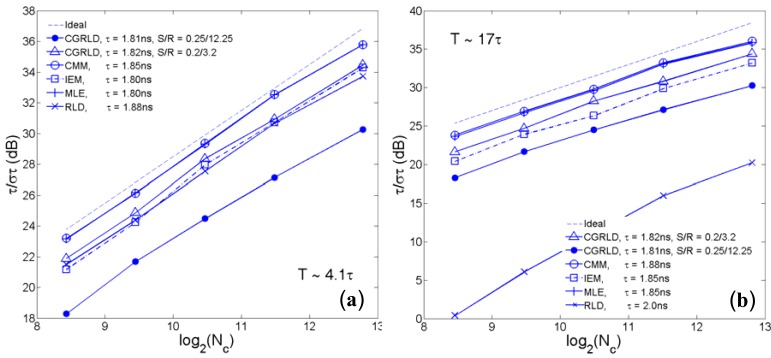
Precision plots for (**a**) Mh = 4.1τ and (**b**) Mh = 17τ.

To compare the error performance of different FLIM algorithms, widefield lifetime images of a uniform aqueous solution of Rhodamine B were taken in different acquisition time. The imaging system was set up on a Nikon TE2000U inverted microscope. The excitation source was a PicoQuant pulsed diode laser with a wavelength of 470 nm coupled through the epi-fluorescence port of the microscope using a Nikon B-2A filter cube. The laser pulse rate is 20 MHz and the maximum power reaching the back focal plane of the objective is about 90 μW. The sample was imaged onto the 32 × 32 SPAD camera directly attached to one of the camera ports using a 20× objective (Nikon Plan Apo, 20×, NA 0.45). The TDC resolution is set to be 78 ps with the on-chip phase-locked loop enabled [22]. Two measurement windows of *Mh* ∼ 4.1*τ* and *Mh* ∼ 17*τ* were chosen to calculate lifetimes. Figure 5(a,b) show the precision curves in terms of photon counts for different algorithms. The ideal curve is the theoretical precision of MLE (or CMM) without considering system non-idealities. CMM and MLE behave almost the same in both plots, and they are about 0.5 ∼ 1.5 dB away from the ideal curve. The first window *Mh/τ* ∼ 4.1 is optimized for RLD-2, where RLD-2, IEM, and CGRLD (*S*/*R* = 0.2/3.2) work equally well. Figure 5(b) shows the reason why we need a fast FLIM algorithm other than RLD-2. The CGRLD with S/R = 0.25/12.25 still performs worse than the CGRLD with S/R = 0.2/3.2 making it inefficient in photon collecting due to low duty cycle operations (optimized window > 17τ). The conclusions drawn from Figure 5(a,b) are in good agreement with the theoretical Figure 3(a,d).

### 2.4. Contrast Capability of Algorithms on Bi-Exponential Decays

Assume a bi-exponential decay *f*(*t*) = *A_1_*exp(−*t*/*τ_1_*) + *A_2_*exp(−*t*/*τ_2_*) with *τ_1_*, *τ_2_* being the lifetimes and *A_1_*, *A_2_* being the pre-scalars. Although the above FLIM algorithms are for single-exponential data, it is worth comparing the average lifetimes produced for bi-decay data:

(9) $$\tau_{\text{ave}}=\left(A_{1}\tau_{1}+A_{2}\tau_{2}\right)/\left(A_{1}+A_{2}\right).$$

**Figure 6.**
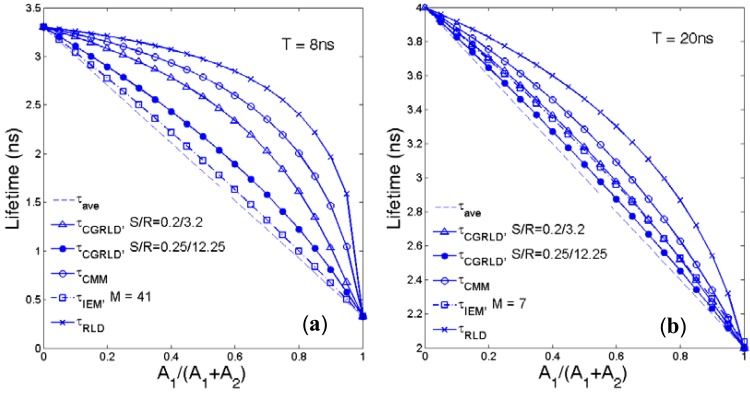
Average lifetime *versus A*_1_/(*A*_1_ + *A*_2_) plots obtained by different methods for (**a**) *τ*_1_ = 0.33 ns, *τ*_2_ = 3.3 ns, *T* = 8 ns and (**b**) *τ*_1_ = 2 ns, *τ*_2_ = 4 ns, *T* = 20 ns.

The average lifetimes defined by IEM [42], RLD-2 [21], CMM [15], and CGRLD are:

(10) $$\begin{array}{l} \tau_{\text{IEM}}=\frac{h\cdot \sum_{j=0}^{M-1}\left({\bar{C}}_{j}N_{j}\right)}{N_{0}-N_{M-1}},N_{j}=\int_{jh}^{\left(j+1\right)h}f\left(t\right)\text{dt}=A_{1}\tau_{1}\left(x_{1}^{j}-x_{1}^{j+1}\right)+A_{2}\tau_{2}\left(x_{2}^{j}-x_{2}^{j+1}\right),\\ \text{or}\;\tau_{\text{IEM}}\sim \frac{\int_{0}^{\left(M-1\right)h}f\left(t\right)\text{dt}}{f\left(0\right)-f\left(\left[M-1\right]h\right)}=\frac{\left[A_{1}\tau_{1}\left(1-x_{1}^{M-1}\right)+A_{2}\tau_{2}\left(1-x_{2}^{M-1}\right)\right]}{\left[A_{1}+A_{2}-A_{1}x_{1}^{M-1}-A_{2}x_{2}^{M-1}\right]}\;\text{if}\;M>>1\;\left[42\right], \end{array}$$

(11) $$\tau_{\text{RLD}-2}=\frac{0.5T}{\ln\left(\frac{N_{1}}{N_{2}}\right)}=\frac{0.5T}{\ln\left(\frac{\int_{0}^{0.5T}f\left(t\right)dt}{\int_{0.5T}^{T}f\left(t\right)dt}\right)}=\frac{0.5T}{\ln\left[\frac{A_{1}\tau_{1}\left(1-e^{-0.5T/\tau_{1}}\right)+A_{2}\tau_{2}\left(1-e^{-0.5T/\tau_{2}}\right)}{A_{1}\tau_{1}\left(e^{-0.5T/\tau_{1}}-e^{-T/\tau_{1}}\right)+A_{2}\tau_{2}\left(e^{-0.5T/\tau_{2}}-e^{-T/\tau_{2}}\right)}\right]},$$

(12) $$\tau_{\text{CMM}}=T\cdot \Omega \left(\frac{\int_{0}^{T}t\cdot f\left(t\right)dt}{T\int_{0}^{T}f\left(t\right)dt}\right)=T\cdot \Omega \left[\frac{A_{1}\tau_{1}^{2}\left(1-x_{1}^{M}-x_{1}^{M}\frac{T}{\tau_{1}}\right)+A_{2}\tau_{2}^{2}\left(1-x_{2}^{M}-x_{2}^{M}\frac{T}{\tau_{2}}\right)}{A_{1}\tau_{1}\left(1-x_{1}^{M}\right)T+A_{2}\tau_{2}\left(1-x_{2}^{M}\right)T}\right],$$

(13) $$\tau_{\text{CGRLD}}=\frac{-h}{\ln\left(x\right)},x{\left(\sum_{i=0}^{R-S-1}x^{i}\right)}^{\frac{1}{S}}=\frac{N_{2}^{\frac{1}{S}}}{N_{1}^{\frac{1}{S}}}={\left(\frac{\int_{Sh}^{Rh}f\left(t\right)dt}{\int_{0}^{h}f\left(t\right)dt}\right)}^{\frac{1}{S}}={\left[\frac{A_{1}\tau_{1}\left(x_{1}^{S}-x_{1}^{R}\right)T+A_{2}\tau_{2}\left(x_{2}^{S}-x_{2}^{R}\right)}{A_{1}\tau_{1}\left(1-x_{1}\right)T+A_{2}\tau_{2}\left(1-x_{2}\right)}\right]}^{\frac{1}{S}},$$

where *x*_1_ = exp(−*h*/*τ*_1_), *x*_2_ = exp(−*h*/*τ*_2_), Ω(·) is the 1-D look up table described in [15], and *h* = *T*/*M* for IEM/CMM and *h* = *T*/(*S* +*R*) for CGRLD. From Equation (10), the average lifetime obtained by IEM will be almost the same with the commonly used Equation (9) when *M* ≫ 1. The conclusion will be the same for CGRLD for *R* ≫ 1. Figure 6 shows the average lifetimes *versus A*_1_/(*A*_1_ + *A*_2_) obtained by Equations (9–13) at (a) *τ*_1_ = 0.33 ns, *τ*_2_ = 3.3 ns, *T* = 8 ns and (b) *τ*_1_ = 2 ns, *τ*_2_ = 4 ns, *T* = 20 ns. In Figure 6(b), IEM-7 and CGRLD (*S*/*R* = 0.2/3.2) have similar performances. In both cases, the RLD curves are furthest away from Equation (9).

**Figure 7.**
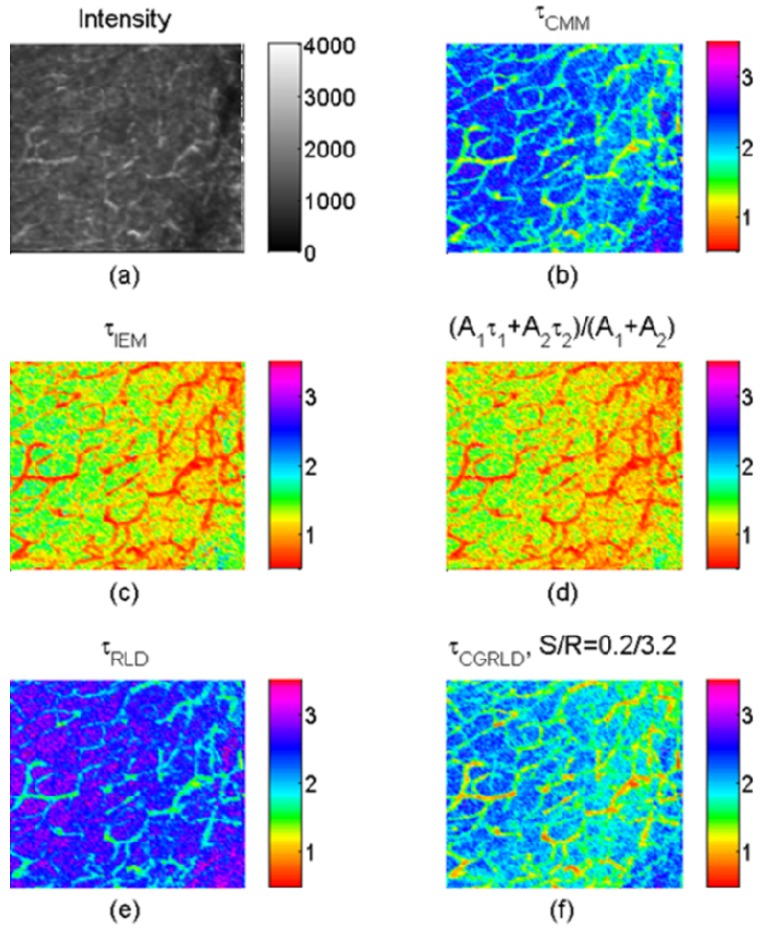
Images of FITC-albumin in a BD9 rat bearing a P22 tumour, 1 hour 40 minutes post administration. (**a**) Intensity image and lifetime images obtained by (**b**) 150-bin CMM; (**c**) 51-bin IEM; (**d**) Equation (9): *τ*_ave_ = (*A*_1_*τ*_1_ + *A*_2_*τ*_2_)/(*A*_1_ + *A*_2_); (**e**) 2-gate RLD; and (**f**) CGRLD with S/R = 0.2/3.2.

**Figure 8.**
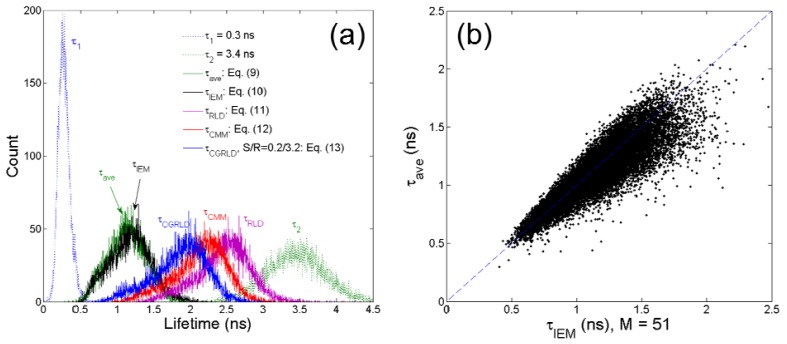
(**a**) Lifetime histograms obtained by different FLIM algorithms and (**b**) *τ*_ave_ *versus τ*_IEM_ plot.

To demonstrate how the above-mentioned single-exponential FLIM algorithms perform on bi-exponential data, we apply them to lifetime imaging data obtained by multi-photon FLIM. The experimental procedure is given in full elsewhere [55]. Briefly, early generation transplants of the P22 rat carcinosarcoma were grown in transparent window chambers, surgically implanted into the fascial layer of a dorsal skin flap of male BD9 rats [56,57]. For imaging of the blood vessel architecture, the molecular probe FITC conjugated Bovine serum albumin was injected intravenously as a blood poolcontrast agent. After 100 minutes the FITC-BSA was observed to diffuse from the vasculature and the FLIM image taken. The intensity image in Figure 7(a) shows very little contrast between the vessels. and the tumor bulk. Compared with the intensity image, the lifetime images Figure 7(b–f) obtained by 150-bin CMM, 51-bin IEM, Equation (9), RLD-2, and CGRLD show much better contrast. The lifetimes in the vessels are smaller than those in the extra-vascular space. Figures 7(c,d) show very similar results except that there is a slightly larger variation in the IEM image shown in Figure 8(b). From Figures 6(a), 7(b) and 7(e), RLD and CMM generate larger lifetimes in the extra-vascular space due to, for example, the *τ*_2_^2^ term in Equation (12). If *A*_1_ is small throughout the image, the RLD and CMM produce less contrast than the other algorithms. They favor a larger *A*_1_ distribution. The image data can also be analyzed with a bi-exponential fit algorithm throughout the image, and the lifetime components are *τ*_1_ = 0.3 ± 0.06 ns and *τ*_2_ = 3.4 ± 0.4 ns. The lifetime histograms for different algorithms are shown in Figure 8(a), and Table 1 shows the calculated lifetimes. Figure 8(b) shows that the lifetimes obtained by Equation (9) are highly correlated with those obtained by the 51-bin IEM. From Figure 8(b), the lifetime histogram showing a slightly larger variation in the IEM data reveals that the bi-exponential algorithms are crucial for examining most biological image data, but we are still benefitting from the high imaging speed and compactness single-exponential models can provide.

## 4. Conclusions

We have reviewed the latest developments on CMOS based FLIM systems and introduced several non-iterative high-speed time-domain algorithms suitable for massive sensor arrays. The theoretical error equations of the FLIM algorithms and their optimized operation conditions are discussed and compared. We have drawn from the theory several conclusions different from those in previously published literature and demonstrated the proposed gating schemes or FLIM algorithms on single-exponential data obtained from 0.13 μm CMOS SPAD arrays. The experimental results show good agreement with the theory. The contrast capabilities of the proposed algorithms on bi-exponential data are also discussed and demonstrated with an *in vivo* two photon fluorescence lifetime imaging data of FITC-albumin labeled vasculature of a P22 rat carcinosarcoma. With emerging CMOS imaging sensors, the proposed techniques capable of rapidly producing lifetime images with enough contrast show great potential in scientific and clinical applications.

## Supplementary Material



## Figures and Tables

**Table 1.** Calculated lifetimes obtained by different algorithms.

**Methods**	**Bi-Decay Model**	Equation (9)	**IEM M = 51**	**RLD**	**CMM**	**CGRLD S/R = 0.2/3.2**
*τ* (ns)	*τ*_1_ = 0.3 ± 0.06	1.13 ± 0.24	1.18 ± 0.28	2.45 ± 0.37	2.13 ± 0.34	1.86 ± 0.36
	*τ*_2_ = 3.4 ± 0.40					

## Appendix

Similar to the analysis methods introduced in [45], we assume that the fluorescence decay function f(t) = Aexp(−t/τ)u(t), with τ being the lifetime and A the prescaler, but here h is the gate width of the “first” gate. Suppose $EN_{c}=\int_{0}^{T=Rh}A\exp\left(-t/\tau \right)dt=A\tau \left[1-e^{-Rh/\tau }\right]=A\tau \left[1-x^{R}\right]$, where *x* = e^−^*^h^*^/^*^τ^* then the expectation values for N_1_ and N_2_ in both categories can be obtained as:

(A1) $$\begin{array}{l} EN_{1}=\int_{0}^{h}A\exp\;\left(-t/\tau \right)dt=A\tau \left(1-e^{-h/\tau }\right)=EN_{c}\left(1-x\right)/\left(1-x^{R}\right)\;\text{and}\;\\ EN_{2}=\int_{Sh}^{Rh}A\exp\;\left(-t/\tau \right)dt=A\tau \left(e^{-Sh/\tau }-e^{-Rh/\tau }\right)=EN_{c}\left(x^{S}-x^{R}\right)/\left(1-x^{R}\right),\text{respectively.} \end{array}$$

We adopt the similar notation as in [45] for the recorded counts *N_j_*, which are Poisson distributed with respective mean values *EN_j_* and standard deviations *σN_j_* = (*EN_j_*)^1/2^, *j* = 1, 2. From Equation (A1), *EN*_1_ and *EN*_2_ are related as *EN*_1_ (*x^S^* − *x^R^*) − *EN*_2_ (1 − *x*) = 0. We define:

(A2) $$g\left(\hat{x}\right)\equiv N_{1}\left({\hat{x}}^{S}-{\hat{x}}^{R}\right)-N_{2}\left(1-\hat{x}\right).$$

Usually, the lifetime can be extracted from the recorded *N*_1_ and *N*_2_ in each pixel by solving *g*(*xˆ*) = 0 and *τˆ* = −*h*/ln(*xˆ*) and the prescaler *Aˆ* = *A* + *σ_A_* can be obtained by solving *Aˆ* =*N_c_*/[*τˆ*(1 − e^−^*^T^*^/^*^τˆ^*]. There is often no experimental interest in the parameter *A* [45], so we concentrate here on the derivation of *σ_x_*. With the variations *σN_j_* in the recorded counts *N_j_* (*j* = 1, 2), the calculated *xˆ* will contain a variation *σ_x_* with respective to its mean value *x* accordingly, *xˆ* = *x* + *σ_x_*. To express *σ_x_* in terms of *σN_j_* for the UGRLD we substitute *N_j_* = *EN_j_* + *σN_j_* (*j* = 1, 2) into Equation (A2) and *g*(*x* + *σ_x_*) = 0, and we can obtain:

(A3) $$\begin{array}{l} \left(EN_{1}+\sigma N_{1}\right)\left[{\left(x+\sigma_{x}\right)}^{S}-{\left(x+\sigma_{x}\right)}^{R}\right]-\left(EN_{2}+\sigma N_{2}\right)\left(1-x-\sigma_{x}\right)=0,\\ \Rightarrow \sigma_{x}\left[EN_{2}+EN_{1}\left(Sx^{S-1}-Rx^{R-1}\right)\right]=\sigma N_{2}\left(1-x\right)-\sigma N_{1}\left(x^{S}-x^{R}\right)\\ =\sqrt{{\left(1-x\right)}^{2}{\left(\sigma N_{2}\right)}^{2}+\left(x^{S}-x^{R}\right){\left(\sigma N_{1}\right)}^{2}}, \end{array}$$

where the higher order terms $\sigma_{x}^{2}$, $\sigma_{x}^{3}$, … are negligible, and the random variables *N_1_* and *N_2_* are independent for the UGRLD.

To derive the *F*-value for the CGRLD, we substitute *N*_1_ = *N*_1″_ + *N*_ov_ = *EN*_1″_ + *σ N*_1″_ + *EN*_ov_ + *σ N*_ov_ and *N*_2_ = *N_2_*_″_ + *N_ov_* = *EN_2_*_″_ + *σ N_2_*_″_ + *EN*_ov_ + *σ N*_ov_ into Equation (A2) with *EN*_1_ = *EN*_1″_ + *EN*_ov_ and *EN*_2_ = *EN*_2″_ + *EN*_ov_:

(A4) $$\begin{array}{l} \left(EN_{1}+\sigma N_{1^{\prime \prime }}+\sigma N_{OV}\right)\left[{\left(x+\sigma_{x}\right)}^{S}-{\left(x+\sigma_{x}\right)}^{R}\right]-\left(EN_{2}+\sigma N_{2^{\prime \prime }}+\sigma N_{OV}\right)\left(1-x-\sigma_{x}\right)=0,\\ \Rightarrow \sigma_{x}\left[EN_{2}+EN_{1}\left(Sx^{S-1}-Rx^{R-1}\right)\right]=\left(\sigma N_{2^{\prime \prime }}+\sigma N_{OV}\right)\left(1-x\right)-\left(\sigma N_{1^{\prime \prime }}+\sigma N_{OV}\right)\left(x^{S}-x^{R}\right)\\ =\sigma N_{2^{\prime \prime }}\left(1-x\right)+\sigma N_{OV}\left(1-x-x^{S}+x^{R}\right)-\sigma N_{1^{\prime \prime }}\left(x^{S}-x^{R}\right)\\ =\sqrt{{\left(1-x\right)}^{2}{\left(\sigma N_{2^{\prime \prime }}\right)}^{2}+{\left(1-x-x^{S}+x^{R}\right)}^{2}{\left(\sigma N_{OV}\right)}^{2}+{\left(x^{S}-x^{R}\right)}^{2}{\left(\sigma N_{1^{\prime \prime }}\right)}^{2}}\\ =\sqrt{{\left(1-x\right)}^{2}EN_{2^{\prime \prime }}+{\left(1-x-x^{S}+x^{R}\right)}^{2}EN_{OV}+{\left(x^{S}-x^{R}\right)}^{2}EN_{1^{\prime \prime }}}\\ =\sqrt{{\left(1-x\right)}^{2}EN_{2}+{\left(x^{S}-x^{R}\right)}^{2}EN_{1}-2\left(1-x\right)\left(x^{S}-x^{R}\right)EN_{OV}} \end{array}$$

where $EN_{OV}=\int_{Sh}^{h}A\exp\left(-h/\tau \right)\text{dt}=EN_{c}\left(x^{S}-x\right)/\left(1-x^{R}\right)$ and the random variables *N_1_*_″_, *N*_ov_ and *N_2_*_″_ are mutually independent for the CGRLD. Differentiating *x* = e^−^*^h^*^/^*^τ^* and from Equations (A3) and (A4), we obtain:

(A5) $$\begin{array}{l} \text{dx}=e^{-h/\tau }{\left(-\frac{h}{\tau }\right)}^{\prime }d\tau =x\frac{h}{\tau^{2}}d\tau \Rightarrow \frac{\sigma_{\tau }}{\tau }=\frac{\tau }{h}\cdot \frac{\sigma_{x}}{x}\\ \Rightarrow \frac{\sigma_{\tau }}{\tau }=\frac{\tau }{h}\cdot \frac{\sqrt{{\left(1-x\right)}^{2}EN_{2}+{\left(x^{S}-x^{R}\right)}^{2}EN_{1}+C\cdot EN_{OV}}}{x\left[EN_{2}+EN_{1}\left(Sx^{S-1}-Rx^{R-1}\right)\right]},C=\{\begin{array}{c} 0\;,\text{for UGRLD}\\ -2\left(1-x\right)\left(x^{S}-x^{R}\right),\text{for CGRLD} \end{array}\\ \Rightarrow \frac{\sigma_{\tau }\sqrt{EN_{c}}}{\tau }=\frac{\tau }{h}\cdot \frac{\sqrt{1-x^{R}}\cdot \sqrt{{\left(1-x\right)}^{2}\left(x^{S}-x^{R}\right)+{\left(x^{S}-x^{R}\right)}^{2}\left(1-x\right)+C\cdot \left(x^{S}-x\right)}}{x\left[x^{S}-x^{R}+\left(1-x\right)\left(Sx^{S-1}-Rx^{R-1}\right)\right]}\\ \Rightarrow \frac{\sigma_{\tau }\sqrt{N_{c}}}{\tau }=\frac{\sigma_{\tau }\sqrt{EN_{c}+\sigma N_{c}}}{\tau }\sim \frac{\sigma_{\tau }\sqrt{EN_{c}}}{\tau }\left(1+\frac{\sigma N_{c}}{2EN_{c}}\right)\\ ≅\frac{\tau }{h}\cdot \frac{\sqrt{1-x^{R}}\cdot \sqrt{{\left(1-x\right)}^{2}\left(x^{S}-x^{R}\right)+{\left(x^{S}-x^{R}\right)}^{2}\left(1-x\right)+C\cdot \left(x^{S}-x\right)}}{x\left[x^{S}-x^{R}+\left(1-x\right)\left(Sx^{S-1}-Rx^{R-1}\right)\right]},\;\text{when}\;\frac{\sigma N_{c}}{2EN_{c}}<<1. \end{array}$$

## References

[1] Medintz I.L., Uyeda H.T., Goldman E.R., Mattoussi H. (2005). Quantum dot bioconjugates for imaging, labelling and sensing. Nat. Mater..

[2] Carlin L.M., Evans R., Milewicz H., Matthews D.R., Fernandes L., Perani M., Levitt J., Keppler M., Monypenny J., Coolen A. (2011). A targeted siRNA screen identifies regulators of Cdc42 activity at the Natural Killer cell immunological synapse. Sci. Signal..

[3] Fruhwirth G.O., Fernandes L.P., Weitsman G., Patel G., Kelleher M., Lawler K., Brock A., Poland S.P., Mattews D.R., Keri G. (2011). How föster resonance energy transfer imaging improves the understanding of protein interaction networks in cancer biology. Chem. Phys. Chem..

[4] Lakowicz J.R., Szmacinski H., Nowaczyk K., Johnson M.L. (1992). Fluorescence lifetime imaging of calcium using Quin-2. Cell Calcium.

[5] Spring B.Q., Clegg R.M. (2009). Image analysis for denoising full-field frequency-domain fluorescence lifetime images. J. Microsc..

[6] Gratton E., Breusegem S., Sutin J., Ruan Q., Barry N. (2003). Fluorescence lifetime imaging for the two-photon microscope: Time-domain and frequency-domain methods. J. Biomed. Opt..

[7] Clayton A.H.A., Hanley Q.S., Verveer P.J. (2004). Graphical representation and multicomponent analysis of single-frequency fluorescence lifetime imaging microscopy data. J. Microsc..

[8] Becker W. (2005). Advanced Time-Correlated Single Photon Counting Techniques.

[9] Yoon H.-J., Itoh S., Kawahito S. (2009). A CMOS image sensor with in-pixel two-stage charge transfer for fluorescence lifetime imaging. IEEE Trans. Electron. Devices.

[10] Huang T.-C.D., Sorgenfrei S., Gong P., Levicky R., Shepard K.L. (2009). A 0.18 μm CMOS array sensor for integrated time-resolved fluorescence detection. IEEE J. Solid-State Circuits.

[11] Pancheri L., Stoppa D. A SPAD-Based Pixel Linear Array for High-Speed Time-Gated Fluorescence Lifetime Imaging.

[12] Benetti M., Iori D., Pancheri L., Borghetti F., Pasquardini L., Lunelli L., Pederzolli C., Gonzo L., Betta G.-F.D., Stoppa D. (2010). Highly parallel SPAD detector for time-resolved lab-on-chip. Proc. SPIE.

[13] Rae B.R., Yang J.B., Mckendry J., Gong Z., Renshaw D., Gerkin J.M., Gu E., Dawson M.D., Henderson R.K. (2010). A vertically integrated CMOS microsystem for time-resolved fluorescence analysis. IEEE Trans. Biomed. Circuits Syst..

[14] Li D.-U., Arlt J., Richardson J., Walker R., Buts A., Stoppa D., Charbon E., Henderson R. (2010). Real-time fluorescence lifetime imaging system with a 32 × 32 0.13 μm CMOS low dark-count single-photon avalanche diode array. Opt. Express.

[15] Li D.D.-U., Arlt J., Tyndall D., Walker R., Richardson J., Stoppa D., Charbon E., Henderson R.K. (2011). Video-rate fluorescence lifetime imaging camera with CMOS single-photon avalanche diode arrays and high-speed imaging algorithm. J. Biomed. Opt..

[16] Veerappan C., Richardson J., Walker R., Li D.-U., Fishburn M.W., Maruyama Y., Stoppa D., Borghetti F., Gersbach M., Henderson R.K. A 160 × 128 Single-Photon Image Sensor with on-pixel 55ps 10bit Time-to-Digital Converter.

[17] Maruyama Y., Charbon E. An All-Digital, Time-Gated 128 × 128 SPAD Array for On-Chip, Filter-Less Fluorescence Detection.

[18] Guo J., Sonkusale S. A CMOS Imager with Digital Phase Readout for Fluorescence Lifetime Imaging.

[19] Yao L., Yung K.Y., Cheung M.C., Chodavarapu V.P., Bright F.V. CMOS Direct Time Interval Measurement of Long-Lived Luminescence Lifetimes.

[20] Tyndall D., Rae B., Li D., Richardson J., Arlt J., Henderson R. A 100Mphoton/s Time-Resolved Mini-Silicon Photomultiplier with On-Chip Fluorescence Lifetime Estimation in 0.13 μm CMOS Imaging Technology.

[21] Ballew R.M., Demas J.N. (1989). An error analysis of the rapid lifetime determination method for the evaluation of single exponential decays. Anal. Chem..

[22] Richardson J., Walker R., Grant L., Stoppa D., Borghetti F., Charbon E., Gersbach M., Henderson R. A 32 × 32 50ps Resolution 10bit Time to Digital Converter Array in 130 nm CMOS for Time Correlated Imaging.

[23] Li L.-Q., Davis L.M. (1993). Single photon avalanche diode for single molecule detection. Rev. Sci. Instrum..

[24] Colyer R.A., Scalia G., Villa F.A., Guerrieri F., Tisa S., Zappa F., Cova S., Weiss S., Michalet X. (2011). Ultra high-throughput single molecule spectroscopy. Proc. SPIE.

[25] Wang Y., Rae B.R., Henderson R.K., Gong Z., Mckendry J., Gu E., Dawson M.D., Turnbull G.A., Samuel I.D.W. (2011). Ultra-portable explosives sensor based on a CMOS fluorescence lifetime analysis micro-system. AIP Adv..

[26] Niclass C., Soga M. A Miniature Actively Recharged Single-Photon Detector Free of Afterpulsing Effects with 6 ns Dead Time in a 0.18 μm CMOS Technology.

[27] Richardson J.A., Grant L.A., Henderson R.K. (2009). Low dark count single-photon avalanche diode structure compatible with standard nanometer scale CMOS technology. IEEE Photon. Tech. Lett..

[28] Henderson R.K., Webster E.A.G., Walker R., Richardson J.A., Grant L.A. A 3 × 3, 5 μm Pitch, 3-Transistor Single Photon Avalanche Diode Array with Integrated 11 V Bias Generation in 90 nm CMOS Technology.

[29] Richardson J.A., Webster E.A.G., Grant L.A., Henderson R.K. (2011). Scalable single-photon avalanche diode structure in nanometer CMOS technology. IEEE Trans. Electron Dev..

[30] Webster E.A.G., Richardson J.A., Grant L.A., Renshaw D., Henderson R.K. (2012). A single-photon avalanche diode in 90-nm CMOS imaging technology with 44% photon detection efficiency at 690 nm. IEEE Electron. Dev. Lett..

[31] Niclass C., Soga M., Matsubara H., Kato S. A 100 m-Range 10-Frame/s 340 × 96-Pixel Time-of-Flight Depth Sensor in 0.18 μm CMOS.

[32] Elangovan M., Day R.N., Periasamy A. (2002). Nanosecond fluorescence resonance energy transfer-fluorescence lifetime imaging microscopy to localize the protein interactions in a single living cell. J. Microsc..

[33] Sharman K.K., Periasamy A. (1999). Error analysis of the rapid lifetime determination method for double-exponential decays and new windowing schemes. Anal. Chem..

[34] Grauw C.J., Gerritsen H.C. (2001). Multiple time-gate module for fluorescence lifetime imaging. Appl. Spectrosc..

[35] Chang C.W., Mycek M.-A. (2010). Enhancing precision in time-domain fluorescence lifetime imaging. J. Biomed. Opt..

[36] Rae B.R., Muir K.R., Gong Z., McKendry J., Girkin J.M., Gu E., Renshaw D., Dawson M.D., Henderson R.K. (2009). A CMOS time-resolved fluorescence lifetime analysis micro-system. Sensors.

[37] Elson D.S., Munro I., Requejo-Isidra J., McGinty J., Dunsby C., Galletly N., Stamp G.W., Neil M.A.A., Lever M.J., Kellett P.A. (2004). Real-time time-domain fluorescence lifetime imaging including single-shot acquisition with a segmented optical image intensifier. New J. Phys..

[38] Agronskaia A.V., Tertoolen L., Gerritsen H.C. (2003). High frame rate fluorescence lifetime imaging. J. Phys. D Appl. Phys..

[39] Agronskaia A.V., Tertoolen L., Gerritsen H.C. (2004). Fast fluorescence lifetime imaging of calcium in living cells. J. Biomed. Opt..

[40] Gerritsen H.C., Asselbergs M.A.H., Agronskaia A.V., Vansark J.H.M. (2002). Fluorescence lifetime imaging in scanning microscopes: acquisition speed, photon economy and lifetime resolution. J. Microsc..

[41] Chan S.P., Fuller Z.J., Demas J.N., DeGraff B.A. (2001). Optimized gating scheme for rapid lifetime determinations of single-exponential luminescence lifetimes. Anal. Chem..

[42] Li D.-U., Bonnist Renshaw D., Henderson R. (2008). On-chip time-correlated fluorescence lifetime extraction algorithms and error analysis. J. Opt. Soc. Am. A.

[43] Li D.-U., Andrews R., Arlt J., Henderson R. (2010). Hardware implementation algorithm and error analysis of high-speed fluorescence lifetime sensing systems using center-of-mass method. J. Biomed. Opt..

[44] Draaijer A., Sanders R., Gerritsen H.C., Pawley J. (1995). Fluorescence Lifetime Imaging, a New Tool in Confocal Microscopy. Handbook of Biological Confocal Microscopy.

[45] Hall P., Selinger B. (1981). Better estimates of exponential decay parameters. J. Phys. Chem..

[46] Li D.-U., Walker R., Richardson J., Rae B., Buts A., Renshaw D., Henderson R. (2009). Hardware implementation and calibration of background noise for an integration-based fluorescence lifetime sensing algorithm. J. Opt. Soc. Am..

[47] Trabesinger W., Hübner C.G., Hecht B., Wild T.P. (2002). Continuous real-time measurement of fluorescence lifetime. Rev. Sci. Instrum..

[48] Moon S, Won Y.J., Kim D.Y. (2009). Analog mean-delay method for high-speed fluorescence lifetime measurement. Opt. Express.

[49] Wong Y.J., Han W.-T., Kim D.Y. (2011). Precision and accuracy of the analog mean-delay method for high-speed fluorescence lifetime measurement. J. Opt. Soc. Am. A.

[50] Won Y.J., Moon S., Yang W.B., Kim D.G., Han W.-T., Kim D.Y. (2011). High-speed confocal fluorescence lifetime imaging microscopy (FLIM) with the analog mean delay (AMD) method. Opt. Express.

[51] Padilla-Parra S., Auduge N., Coppey-Moisan M., Tramier M. (2008). Quantitative FRET analysis by fast acquisition time domain FLIM at high spatial resolution in living cells. Biophys. J..

[52] Yamada H., Padilla-Parra S., Park S.-J., Itoh T., Chaineau M., Monaldi I., Cremona O., Benfenati F., Camilli P.D., Coppey-Moisan M. (2009). Dynamic interaction of amphiphysin with N-WASP regulates actin assembly. J. Biol. Chem..

[53] Barber P.R., Ameer-Beg S.M., Pathmannanthan S., Rowly M., Collen A.C.C. (2010). A Bayesian method for single molecule fluorescence burst analysis. Biomed. Opt. Express.

[54] Rowley M.I, Barber P.R., Coolen A.C.C., Vojnovic B. (2011). Bayesian analysis of fluorescence lifetime imaging data. Proc. SPIE.

[55] Ameer-Big S.M., Barber P.R., Hodgkiss R.J., Locke R.J., Newman R.G., Tozer G.M., Vojnovic B., Wilson J. (2002). Application of multiphoton steady state and lifetime imaging to mapping of tumour vascular architecture. in vivo. Proc. SPIE.

[56] Papenfuss H.D., Gross J.F., Intaglietta M., Treese F.A. (1979). A transparent access chamber for the rat dorsal skin fold. Microvasc. Res..

[57] Dewhirst M.W., Gustafson C., Gross J.F., Tso C.Y. (1987). Temporal effects of 5.0 Gy radiation in healing subcutaneous micro-vasculature of a dorsal flap window chamber. Rad. Res..

